# Tomotherapy as a tool in image-guided radiation therapy (IGRT): current clinical experience and outcomes

**DOI:** 10.2349/biij.3.1.e17

**Published:** 2007-01-01

**Authors:** S Yartsev, T Kron, J Van Dyk

**Affiliations:** 1 Physics and Engineering, London Regional Cancer Program, London Health Sciences Centre, London, Ontario, Canada; 2 Department of Physical Sciences, Peter MacCallum Cancer Centre, East Melbourne, Australia; 3 Departments of Oncology and Medical Biophysics, University of Western Ontario, London, Ontario, Canada

**Keywords:** Image guidance, helical tomotherapy, radiation therapy, IGRT

## Abstract

Modern radiotherapy is characterised by a better target definition through medical imaging accompanied by significantly improved radiation delivery methods, most notably Intensity-Modulate Radiation Therapy (IMRT). However, the treatment can only be as accurate as the positioning of patients for their daily radiotherapy fraction. It is in this context that a number of imaging modalities - ranging from ultrasound to on-board kilovoltage imaging and computed tomography (CT) - have found their way into the treatment room where they verify accurate patient positioning prior to or even during delivery of radiation. Helical tomotherapy (HT) combines IMRT delivery with in-built image guidance using megavoltage CT scanning. This paper discusses the initial experience of different centres with IGRT using HT illustrated by a number of clinical examples from the installation in London in Ontario, Canada, one of the world’s first HT sites. We found that HT allows the delivery of highly conformal radiation dose distributions combined with adequate daily image acquisition. An important feature of this unit is its seamless integration, which also includes a customised inverse treatment planning system and a quality assurance module for individual patients.

## INTRODUCTION

Clinical experience is always a crucial component in the evaluation of any new technology in radiation therapy and helical tomotherapy (HT) is no exception. In the early implementation stages, the attention was focused on retrospective comparisons [1-9] of treatment plans developed for different radiation delivery options in search of clinical scenarios where HT is able to offer a significant improvement due to its specific technological design, as discussed in the preceding review [10]. These plan comparisons concluded that indeed HT can provide improved normal tissue sparing and highly conformal target coverage. Image-guided radiation therapy (IGRT) available on HT, thanks to on-board megavoltage computed tomography (MVCT) implemented in the commercially available Hi-ART model, allows daily patient setup verification and repositioning. In this report, the first results on its use in phantom studies and clinical practice are reviewed.

## CLINICAL EXAMPLES OF IMAGE GUIDANCE IN TOMOTHERAPY

MVCT was found to be an important imaging tool for precise radiation delivery because it provides considerably more anatomical detail than conventional radiation therapy port films used for patient setup verification. There is a growing number of publications comparing treatment plans of different radiotherapy techniques to HT delivery. The latter is predicted to have some advantages, especially concerning homogeneity of the dose distribution in the target [4,11-15]. In the following, we would like to illustrate the specific characteristics of HT treatment plans using clinical examples from our practice in London, in Ontario, Canada. Lung cancer was chosen since the treatment outcomes are quite bad and there is an indication that dose escalation may improve clinical outcomes [16]. In order to achieve this, the dose to normal lung must be reduced. This is a significant challenge in radiation dose delivery. Head and neck, and prostate, the two other examples chosen, are the most common applications for IMRT. Radiotherapy is often the primary treatment modality in these diseases and in both cases, it has been shown that normal tissue toxicity can be reduced by using advanced radiotherapy techniques.

### Lung cancer

Planning studies on the use of HT for treating localised lung cancer showed more conformal dose distribution for the target and better sparing of normal structures [2,17,18]. It would result in a better clinical outcome for radiation treatment if the patient setup, target shape, size and location remain the same as at the time when this patient was imaged for planning. If these conditions are not met, a more conformal dose distribution might partially miss the target and deliver high dose to the sensitive organs. Several imaging techniques have demonstrated significant variations of the tumour volume during radiotherapy treatment: electronic portal imaging showed tumour shrinkage of 20% or more in 40% of patients [19], repeat kilovoltage CT studies for 40 patients revealed a time trend towards decreasing gross tumour volumes (GTVs) during fractionated stereotactic radiotherapy [20], and MVCT on tomotherapy system allowed daily volumetric evaluation [21]. These findings indicate that periodic adjustments of treatment plans during a treatment course are needed to account for changes in shape and location of the target volume and critical structures when highly conformal techniques such as IMRT are used. A pilot feasibility trial of 10 patients with non-small-cell lung cancer provided results on contouring targets on tomotherapy MVCT and conventional CT images [22]. The volumetric agreement between conventional CT and MVCT was excellent in 5 out of 7 patients with lesions located primarily in the lung parenchyma while it was suboptimal for primary mediastinal disease. Kupelian *et al*. [21] reported their study of tumour regression during external beam radiotherapy for 10 patients with non-small-cell lung cancer. This tumour reduction study using on-board MVCT on tomotherapy system gave full volumetric evaluation, which was not possible by observations made on portal images obtained during the course of treatment [19]. MVCT scans of the targeted areas were performed multiple times during treatment. The frequency of scanning was determined by the treating physicians so that a total of 274 MVCT scans were obtained on the 10 patients in the range of 9 to 35 scans per patient. Tumour volumes were determined within the treatment planning system, and not by any manual method. For all 10 tumours, the average decrease in volume was 1.2% per day with a range of 0.6% to 2.3% per day. The lowest rate of shrinkage was observed for the smallest lesion with an initial volume of 5.9 cm^3^. The highest rate was observed in the largest lesion with an initial volume of 737 cm^3^. Other factors such as histology, level of necrosis and dose-per-fraction may play a role in tumour size reduction [23]. Direct evaluation of tumour regression using MVCT immediately before treatment indicates a potential necessity for plan updating during the treatment [24]. If tumour shrinkage during radiation treatment is clinically significant, treatment plan re-optimisation should be considered, so that the dose to the target remains as initially prescribed and improved sparing of sensitive structures (such as normal lung volume) can be achieved [2]. The clinical significance of ‘plan updating’ (or adaptive radiotherapy) remains to be demonstrated and the correlation of tumour regression with clinical outcomes should be studied [25]. Another retrospective study of tumour regression during treatment on a HT unit of 25 patients with lung cancer showed partial response in 3 (12%), marginal response in 5 (20%) and stable disease in 17 (68%) patients [26]. Tumour regression of more than 25% was observed in 10 patients (40%). However, the authors questioned the clinical significance of this regression and field reductions during radiotherapy because there was no way to document histological disease clearance. In our opinion, a follow-up for a sufficiently long period of time after treatment may be a way of answering this question.

**Figure 1:**
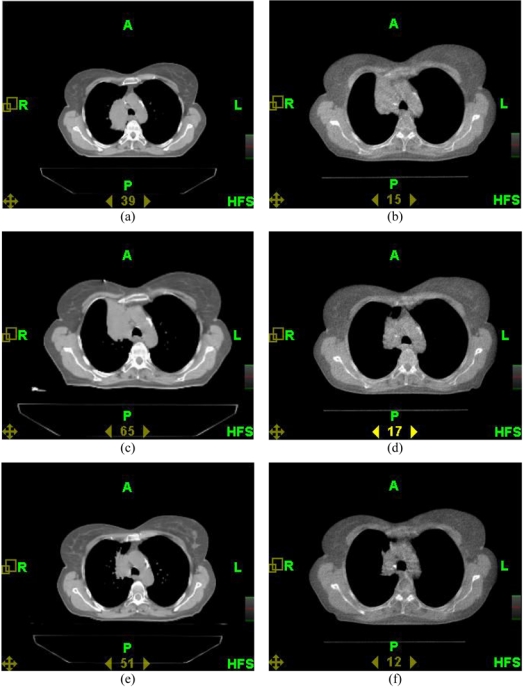
Screenshots of images of the patient with lung cancer for (a) initial kVCT image done 35 days before the treatment start, (b) MVCT image taken 13 days before the treatment start, (c) second MVCT image done 4 days before the treatment start, (d) MVCT image taken before fraction 18 on day 26 of the treatment, (e) third kVCT image done on day 33 of the treatment, (f) MVCT image taken on the last (52nd) day of the treatment, fraction 30.

There are some cases of dramatic anatomy changes revealed by MVCT imaging where a re-planning is absolutely necessary. Figure 1 demonstrates one such example of a patient with non-small-cell lung cancer treated on tomotherapy unit at our centre in 2005. Figure 1a shows one axial slice of the kVCT study used for the initial treatment plan #1. However, when the patient came for treatment 22 days after this study, the MVCT indicated (see Figure 1b) that the tumour had displaced both anteriorly and superiorly probably as a result of a collapse of one of the lung segments. Another kVCT study was performed (see Figure 1c), which confirmed the findings on MVCT and was used for creating plan #2. This plan was delivered for 18 fractions and daily MVCT images showed only slight reduction of the GTV until the day when the atelectasis was resolved and the tumour moved back as shown in Figure 1d. Then yet another kVCT study was performed as shown in Figure 1e; it was used for plan #3. The latter was applied for the remaining fractions and the MVCT image on the final day of treatment is shown in Figure 1f. Clearly, such dramatic changes in tumour/normal tissue anatomy can only be detected by daily CT imaging of the patient.

### Head & neck

**Figure 2:**
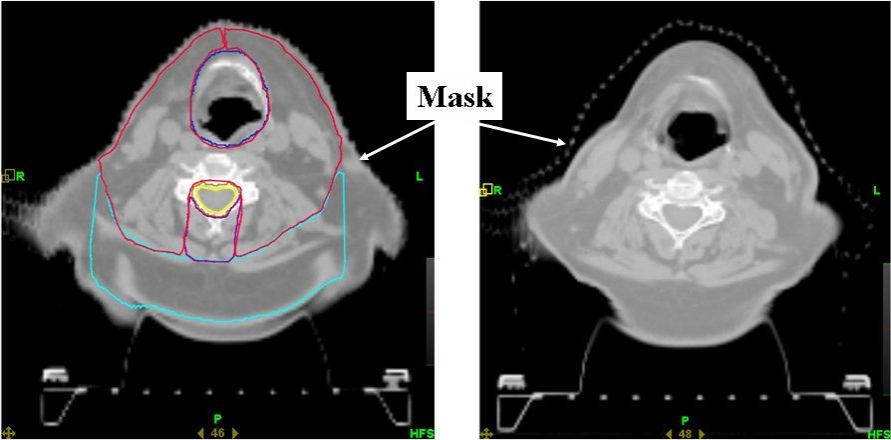
kVCT images (left) before the treatment and (right) after delivery of 22 fractions for the patient with significant weight loss.

**Figure 3:**
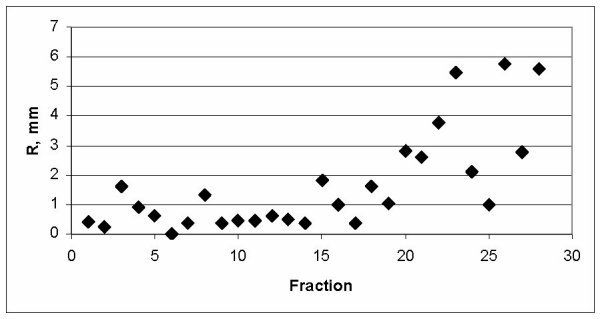
The values of vector shifts introduced daily based on matching of the PTV contour used for planning (red line on Fig. 2a) with the daily MVCT images.

Among the tumour sites, head and neck represent specific challenges and opportunities for high-precision radiotherapy planning and delivery. The proximity – in most cases – between the clinically manifest GTV and the critical organs at risk and the fact that internal motion of tissues and organs tends to be less of an issue in the head and neck region favors the use of high precision IMRT [27]. Hansen *et al*. have found that repeat CT imaging and re-planning during the course of IMRT for patients with head and neck cancer is essential because the clinical target volume decreased at a median rate of 1.7%-1.8% per treatment day and the volume loss was frequently asymmetric [28]. Figure 2 illustrates the importance of daily setup corrections in the case of the patient shown in Figure 3 from our companion paper [10]. A dose of 60 Gy to 90% of the planning target volume (PTV) was prescribed with priority of sparing spinal cord and trachea. This patient lost weight during treatment (from 133 kg at the start to 124 kg at the end of treatment), resulting in changed patient anatomy especially in the treatment area. The vector shifts used for daily patient setup alignment for this patient as a function of time are shown in Figure 3

Re-treatment of spinal metastasis is extremely difficult because the spinal cord typically receives a radiobiological equivalent dose of 40 to 45 Gy given in 2 Gy fractions during the first course of treatment, so that the risk of radiation myelitis after a second course is high [29]. Mahan *et al*. evaluated a feasibility of image-guided tomotherapy for re-treatment of the vertebral spine [30]. They performed measurements of dose gradients and maximum cord doses using a cylindrical phantom, tested the ability of MVCT images to localize spinal anatomy and used this experience for re-treatment of 8 patients with cord compressions to a mean dose of 28 Gy using HT. The total imaging system error was measured by repeat imaging of an anthropomorphic head phantom. At first, kVCT images were acquired on a CT simulator and transferred to the tomotherapy database for image fusion. The phantom was placed in the correct position relative to the machine isocenter, so that any non-zero setup shifts calculated by the image guidance system represent error in the imaging and fusion process. MVCT images were acquired over a 15 cm range superior-inferior and different options of the automatic image fusion algorithm were used. Total imaging system errors (1 σ) of ±0.6 mm, ±0.5 mm, and ±0.6 mm were obtained by "Bone", "Bone and Soft Tissue", and "Full-image" options, respectively. The uncertainty in the superior-inferior direction (±0.6 mm) was twice the uncertainty in the anterior-posterior and lateral directions (±0.3 mm).

It should be noted that the total clinical error may be much larger because real patients can move during treatment and/or the vertebral column can align in a different position relative to the treatment plan. In radiotherapy of 8 patients with previously treated vertebral metastasis, Mahan *et al*. acquired MVCT images through the PTV, autofused them with the planning kVCT images, displaced the patients according to the calculated MVCT/kVCT shifts and again performed MVCT study to verify that the shifts were correctly applied and to assess intrafraction motion before treatment delivery. The range of the total interfraction displacement with respect to the positioning on external laser marks was as great as 15 mm [30]. The standard deviation was 4.0 mm, 4.1 mm, and 4.3 mm in the anterior-posterior, lateral and superior-inferior directions, respectively. Dose gradients of 10% per mm were found achievable by HT in phantom measurements for a geometry representing the spinal cord as a 10 mm diameter cylinder and a 25 mm thick ‘vertebrae’. A very small 3 mm margin was used for expanding the GTV to the PTV assuming high accuracy of positioning and high dose gradients. As a result of such treatment 6 out of 8 patients had complete relief of their pre-treatment symptoms, 2 had partial relief, and 2 died of distant disease during the mean follow-up of 15.2 months [30].

### Prostate

A group from Milan reported their estimates of systematic and random set-up errors by using on-line as well as off-line setup correction protocols. They have modeled the average systematic error and the residual error for the case of post-operative prostate cancer (hypofractionated schedule 20 Gy in 2.9 Gy/fraction) and the minimum number of treatment sessions necessary to correctly estimate systematic set-up error [31]. Another study of effectiveness of daily prostate registration on a tomotherapy unit was done by K. Langen *et al*. [32] using 120 alignments from 3 patients with implanted fiducial markers. They retrospectively compared manual registration (i.e., visual matching of the prostate kVCT image used for planning and current MVCT image) by different techniques. The reference alignment was calculated based on the fiducial markers’ centre of mass. If three implanted solid gold markers clearly visible both in kVCT and MVCT were used, the relative number of alignments, which differed by more than 3 mm from the reference, were 3%, 6% and 3% in the anterior-posterior, superior-inferior and lateral directions, respectively. If gold markers were disregarded and registration was done for the prostate as seen on the MVCT with the prostate as seen on the planning kVCT scan, the respective values for the same deviations were 24%, 33% and 3%. No alignments differed more than 5 mm by this anatomy-based technique. If the organ contours from the plan (the kVCT images as such were not used in this case) were used for registration with the prostate MVCT image, there were more deviations from the reference case by more than 3 mm: the respective values were 55%, 48% and 21%. The anatomy-based registrations outperformed the contour-based registration both in terms of agreement with a marker-based centre-of-mass reference alignment and inter-user variability.

**Figure 4:**
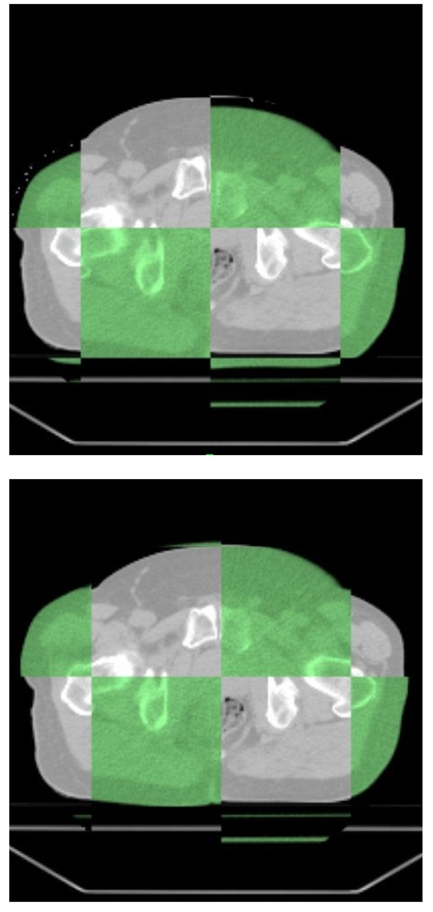
Image showing both kVCT (grey) and MVCT (green) images taken (top) before and (bottom) after registration.

**Figure 5:**
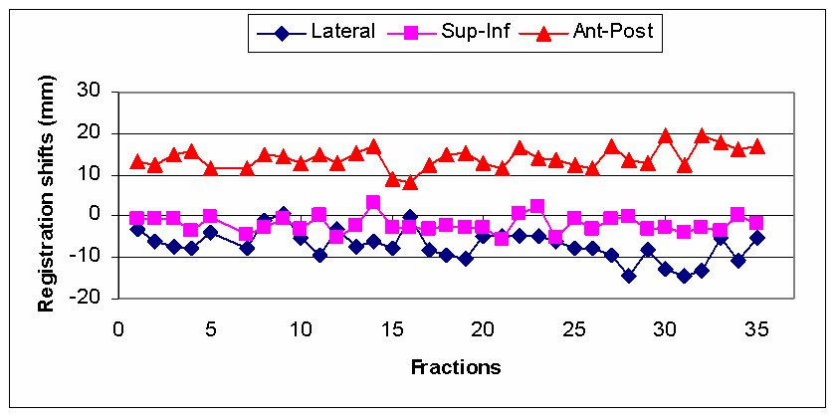
Daily shifts in different directions after registration of the patient with prostate cancer. The adjustments in anterior/posterior direction have a systematic shift due to different mechanical properties of the couches in the diagnostic CT scanner and the tomotherapy unit.

The prostate gland can be identified with sufficient contrast on MVCT images as shown for the patient treated in our institution on images taken before and after registration with diagnostic kVCT study (Figure 4). Daily registration shifts for this patient are shown in Figure 5 for anterior/posterior, lateral and superior / inferior directions. The couches (‘patient support assembly’) on diagnostic CT and tomotherapy unit have different mechanical properties, so a heavier patient with the same initial setup made on external marks is in a lower position on the more flexible couch on tomotherapy. This is detected, taken into account and corrected by MVCT imaging.

Song *et al*. evaluated the image-guidance capabilities of MVCT by comparing volumes of prostate contours outlined on MVCT and kVCT studies of the same 5 patients [33]. Seven observers did the contouring twice with an interval of 2 months, which allowed the evaluation of inter- and intraobserver variability. The volumes defined on the kVCT were smaller and more consistent compared with the MVCT results (54.1 ± 8.6_rms_ cm^3^ and , 59.9 ± 14.8_rms_ cm^3^, respectively). On average, the increase in the clinical target volume variability Δσ = √(σ^2^_MVCT_ - σ^2^_kVCT_), for both interobserver and intraobserver studies was 0.32 cm [33]. This outcome is not surprising, because observers tend to segment larger volumes on images with lower soft-tissue contrast [34,35]. Song et al. suggested that the techniques that do not require contours for deformable-image registration, such as intensity-driven dose-warping techniques [36,37] may be more suitable for MVCT.

When imaging the thorax or abdomen of a patient, respiration-induced artefacts such as blurring, doubling, streaking and distortion degrade the image quality and affect the target localisation ability [38]. These artefacts depend on the ratio between breathing cycle and the gantry rotation speed. In conventional CT or a modern CT scanner, each rotation of the scan can be completed within 1 s or less, during which the organ motion is relatively small. A single slice of MVCT in HT is reconstructed from a 180^o^ rotation in 5 s, which will introduce motion artefacts from breathing. However, other competing techniques suffer from the same problem, e.g., a cone-beam CT scan takes typically 45 s to 1 min for acquiring the projection data in a full 360^o^ scan [39]. Some investigations of motion artefacts in tomotherapy imaging were done [40-42] and it can be expected that future developments will allow for faster image acquisition. Several groups have also worked on gated HT delivery [43,44]. It will be interesting to see how similar approaches can be utilised for improved MVCT acquisition in the future.

## CONCLUSION

Clinical experience with HT is rapidly growing, stimulated by encouraging dosimetric results from planning studies of this method in comparison with traditional techniques. Preliminary results of implementation of IGRT in a tomotherapy setting shows that the on-board MVCT image acquisition system allows improved patient positioning. Increased setup precision permits the use of smaller margins around targets and organs at risk. Clinical experience in different institutions has proved the usefulness of MVCT imaging for corrections of patient setup leading to the possibility of better tumour control and a better sparing of healthy tissues. IGRT benefits individual patients and also, by combining information from many patients, allows radiotherapy departments to develop rational strategies for margin design and the identification of potential weaknesses in the treatment chain.

## ACKNOWLEDGEMENTS

This study was conducted with the support of the Ontario Institute for Cancer Research through funding provided by the government of Ontario.
